# Role of Imaging Techniques in Ovarian Cancer Diagnosis: Current Approaches and Future Directions

**DOI:** 10.3390/cancers18010173

**Published:** 2026-01-04

**Authors:** Alessandro D’Amario, Roberta Ambrosini, Alessandro Gullino, Luigi Grazioli

**Affiliations:** 1 Department of Diagnostic Imaging, ASST Spedali Civili di Brescia, P.le Spedali Civili, 1, 25123 Brescia, Italy; roberta.ambrosini@asst-spedalicivili.it (R.A.);; 2 Department of Medical and Surgical Specialties, Radiological Sciences and Public Health, ASST Spedali Civili di Brescia, Università degli Studi di Brescia, P.le Spedali Civili, 1, 25123 Brescia, Italy

**Keywords:** ovarian cancer, Ultrasound (US), Computed Tomography (CT), Magnetic Resonance Imaging (MRI), O-RADS MRI Score, Artificial Intelligence (AI), radiomics

## Abstract

**Background**: Ovarian cancer is a leading gynecological malignancy with high global mortality. Early and accurate diagnosis is critical for optimal management; however, a considerable portion of ovarian masses remain indeterminate after initial evaluation. Although transvaginal ultrasound is the first-line imaging tool, up to 30% of cases yield inconclusive findings, complicating treatment decisions. **Methods**: This review summarizes current diagnostic strategies for ovarian masses, with an emphasis on advanced imaging and emerging technologies. Topics include the diagnostic utility of contrast-enhanced MRI, the application of the O-RADS MRI scoring system, and the integration of Artificial Intelligence (AI) into imaging workflows. **Results**: Contrast-enhanced MRI offers high diagnostic accuracy (83–93%) for characterizing indeterminate ovarian masses. The O-RADS MRI Score offers a reported sensitivity of 93% and specificity of 91% for malignancy risk assessment. Additionally, new classification systems have been proposed to further improve diagnostic performance. AI-based approaches, particularly machine learning and deep learning applied to imaging data, show potential in improving diagnostic precision; however, most techniques require further clinical validation before widespread adoption. **Conclusions**: Advanced imaging techniques and AI-driven methods are reshaping the diagnostic landscape of ovarian cancer. While current tools like MRI and O-RADS enhance accuracy, ongoing research into novel models and AI applications suggests further improvements are possible. Clinical validation and expert oversight remain essential for their integration into routine practice.

## Simple Summary

Ovarian cancer is a leading cause of death among gynecological malignancies. Standard ultrasound scans may not be conclusive, especially when ovarian masses are difficult to classify. This review highlights recent advances aimed at reducing diagnostic uncertainty. Contrast-enhanced MRI has demonstrated high accuracy in differentiating benign from malignant lesions, and the O-RADS MRI scoring system provides structured risk assessment with strong sensitivity and specificity. New classification methods are also being developed to further support clinical decision-making. In addition, artificial intelligence (AI) approaches, including machine learning and deep learning, are being tested to improve diagnostic precision by analyzing complex imaging data. Overall, the integration of advanced imaging with AI has the potential to substantially improve the evaluation and management of women with suspected ovarian cancer.

## 1. Introduction

In 2020, ovarian cancer (OC) ranked eighth on the list of the most diagnosed malignant diseases worldwide, affecting approximately 314,000 women, moreover being eighth in the overall ranking of cancers most strongly associated with mortality, with more than 207,000 deaths reported [1]. Recent studies suggest a rising incidence of gynecological cancers overall [2]. High-grade serous carcinoma is the most prevalent malignant subtype of OC, representing approximately 70% of cases. The prognosis of ovarian cancer is generally poor due to several factors: its inherently aggressive nature, the absence of specific early signs and symptoms, and the lack of effective population-wide screening programs. As a result, 70–80% of cases are diagnosed at an advanced stage—Stage III or IV according to the 2021 FIGO (International Federation of Gynecology and Obstetrics) staging system—. Consequently, the overall 5-year survival rate is estimated at approximately 46%, making ovarian cancer one of the most lethal malignancies [1]. Staging is surgical and follows the FIGO classification system. The standard treatment approach includes cytoreductive surgery followed by platinum-taxane chemotherapy. Neoadjuvant chemotherapy is administered when primary surgery is not feasible. Targeted therapies such as PARP inhibitors (for BRCA-mutated tumors) and bevacizumab (anti-angiogenic) have improved outcomes. HIPEC and immunotherapy are under evaluation. Radiotherapy plays a limited role, mainly in palliative settings [3,4,5]. In this context imaging plays a pivotal role in the diagnosis, staging, treatment planning and follow-up of ovarian cancer. Given that clinical presentation is often nonspecific, imaging modalities are essential not only for detecting adnexal masses but also for characterizing their nature and distinguishing benign from malignant ovarian lesions. Furthermore, imaging contributes strategically to treatment planning, as the choice between primary cytoreductive surgery and neoadjuvant chemotherapy is largely informed by disease stage and tumor resectability. Hence, advanced imaging techniques and standardized reporting systems have become indispensable tools for radiologists and gynecologic oncologists; in particular, assessment of suitability for primary debulking surgery within multidisciplinary tumor boards constitutes an essential component of treatment planning [6]. The development of standardized imaging systems, such as O-RADS MRI, represents a significant step toward improving diagnostic accuracy and interobserver reproducibility. This review provides an overview of current diagnostic approaches for ovarian masses, with a particular emphasis on advanced MRI, the O-RADS MRI scoring system, and emerging technologies such as Artificial Intelligence (AI), while also outlining future directions in ovarian cancer imaging.

## 2. Imaging in Ovarian Carcinoma

### 2.1. Ultrasonographic Imaging

Ultrasound represents the primary imaging modality for the evaluating and characterizing adnexal masses in both asymptomatic individuals and patients at increased risk of malignancy. The diagnostic performance of ultrasonography—specifically its sensitivity and specificity—depends on several factors, including the operator expertise, the clinical setting in which the examination is performed, the technical quality of the equipment, and the intrinsic features of both the patient and the lesion. Reported values of sensitivity and specificity in the literature are variable, generally ranging from 70% to 90% and from 70% to 95%, respectively [7,8].

Ultrasonography relies on the morphologic features of ovarian lesions, which, when assessed in conjunction with menopausal status and CA-125 levels, allow an estimate of malignancy risk. Operator proficiency, along with the reputation and resources of the investigative centre, plays a crucial role in ensuring diagnostic accuracy and optimal patient outcomes. For this reason, efforts over the years have focused on developing risk-stratification models for ovarian lesions that would be simple to apply, reproducible, and effective. Several approaches have been proposed, including the Risk of Malignancy Index (RMI), Risk of Ovarian Malignancy Algorithm (ROMA) and Logistic Regressions 1 and 2 (LR1 and LR2). More recent contributions to this field include the ADNEX model and the ORADS-US classification system.

#### 2.1.1. IOTA ADNEX Model

The IOTA-ADNEX (International Ovarian Tumor Analysis-Assessment of Different Neoplasias in the Adnexa) model was published in 2014 with the goal of classifying ovarian lesions on ultrasound, and it proved to be more effective than previously used models. The ADNEX model aims not only to predict the likelihood of malignancy (benign vs. malignant) but also to distinguish between different stages of disease progression (borderline, stage I, stage II–IV, metastatic). This capability supports earlier detection of suspicious lesions and, consequently, contributes in reduction in mortality and morbidity [9]. The model takes into account nine variables: patient age (years), serum CA-125 levels (U/mL), type of clinical center (reference/non-reference in oncology), maximum lesion diameter (mm), number of papillary projections (0, 1, 2, 3 or >3), maximum diameter of the largest solid component (mm), presence of more or less than 10 cystic loculi (yes/no) and ascitic effusion (yes/no). The model outputs both numerical and graphical estimates of malignancy risk (%), with studies showing that the ADNEX model achieves high specificity in discriminating between benign and malignant adnexal masses [10].

#### 2.1.2. O-RADS US

The Ovarian-Adnexal Reporting and Data System (O-RADS) US represents the most recent stratification and management system for ovarian and adnexal lesions based on ultrasound features of the lesions. Published in 2018, it aims to establish a common language to minimize ambiguity and to improve the interpretation of ovarian masses [7,11,12]. According to a meta-analysis of Zhang et al. [13], the pooled sensitivity and specificity of O-RADS US were 95% (95% CI, 91–97%) and 82% (95% CI, 76–87%), respectively. Furthermore, it shows substantial inter-observer agreement among radiologists with different level of experience in the risk stratification of ovarian masses [14,15]. Several retrospective external validation studies have also confirmed the diagnostic validity and robustness of the O-RADS US system [14,16].

The O-RADS US system distinguishes “physiologic” findings, such as follicles and corpora lutea, from non-physiological findings, which are classified as “lesions”, both benign and malignant ones. O-RADS US is based on a standardized lexicon that enables the classification of ovarian lesions into categories, each corresponding to a specific risk of malignancy:

- O-RADS 0: technically inadequate/not applicable
- O-RADS 1: physiological findings; no ovarian lesions
- O-RADS 2: lesions almost certainly benign (<1% risk of malignancy)
- O-RADS 3: lesions with low risk of malignancy (1–10%)
- O-RADS 4: lesions with intermediate risk of malignancy (10–50%)
- O-RADS 5: lesions with high risk of malignancy (>50%)

**Figure 1:**
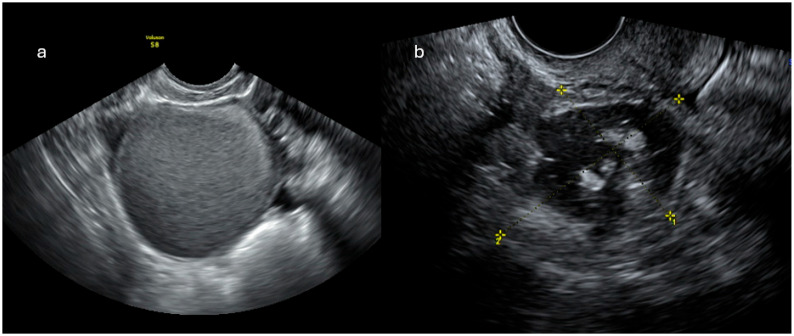
Ultrasound demonstrates a unilocular cyst with a “ground-glass” appearance, consistent with an endometrioma (**a**), and a lesion containing intralesional fat, typical of a dermoid (**b**); both representing classic benign ovarian lesions: O-RADS US score 2 (Courtesy of Dr. Gilda Di Paolo).

Categorization is done by evaluating features such as size (maximum lesion diameter), margins (smooth/irregular), fluid contents (anechoic/hyperechoic/hypoechoic), number of loculi (uni-/bi-/multilocular), solid components (shadowing or not), color-score (1–4 at echo-colordoppler evaluation), number of papillary projections, ascites and peritoneal implants (Table 1, Figure 1).

### 2.2. CT Imaging

**Figure 2:**
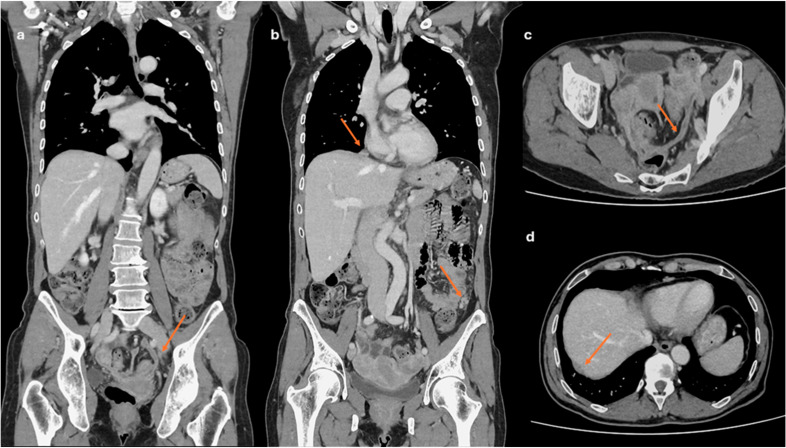
Venous-phase CT staging of ovarian cancer (OC): multiplanar reconstructions (MPR) are mandatory for detecting peritoneal disease (arrow) in the pelvis (**a**,**c**), the subphrenic space (**d**), and along the paracolic gutter ((**b**), lower arrow). CT is also necessary for detection of suspicious lymph nodes ((**b**), upper arrow).

The role of CT imaging is limited in the characterization of adnexal masses due its poor soft tissue resolution in the pelvis; however, contrast-enhanced CT (CE-CT) represents the gold-standard imaging-technique in loco-regional and especially in distant staging of disease [18]. This imaging method boasts of wide availability, relatively low cost and short acquisition time. The CT acquisition protocol involves obtaining scans of 1–2 mm thickness of the neck, thorax, abdomen and pelvis after the injection of iodinated contrast medium. Venous phase images should be acquired 70–80 s after contrast injection, followed by MPR in both the coronal and sagittal planes (Figure 2). There is still no consensus on the use of oral contrast medium administered with the goal of better evaluation of bowel loops and peritoneal carcinosis implants. Oral contrast medium can be positive (iodinated contrast medium) or negative (water).

As stated in the ESUR guidelines, CT should be performed as the initial imaging assessment in patients with advanced ovarian cancer prior to debulking surgery. When administration of iodinated contrast is not feasible or during pregnancy, an alternative approach combining chest CT with abdominal and pelvic MRI can be considered [19].

CT examination allows radiologists to assess the relationships between the mass and surrounding structures (although with less specificity than with MRI), and to identify peritoneal localizations of disease as well as lymph node and parenchymal metastases in both abdominal and extra-abdominal sites. CE-CT has a reported staging accuracy for ovarian cancer of up to 94%, enabling better prediction of the likelihood of successful surgical cytoreduction. In particular, radiological Peritoneal Cancer Index (PCI) correlates with the probability of post-operative residual disease in patients who undergo primary cytoreductive surgery [20,21].

**Figure 3:**
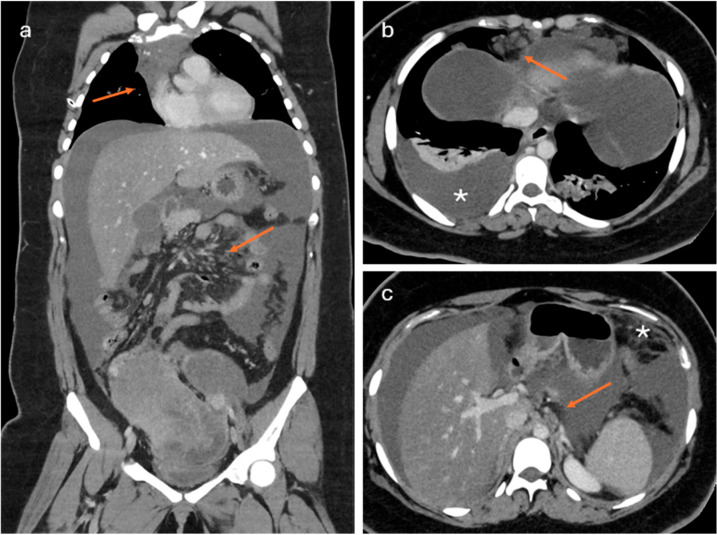
A large adnexal mass in observed in pelvis with peritoneal and pleural effusion ((**b**), asterisk), greater omentum implants (asterisk in (**c**)) and mesentery root involvement (lower arrow in (**a**) and arrow in (**c**)). See also supradiaphragmatic lymph nodes enlargement (upper arrow in (**a**,**b**)). The patient is not eligible for primary surgical treatment.

It is essential to identify and describe all peritoneal localizations, firstly to assess the operability (or not) of the patient, and then for proper surgical planning. The most recent ESUR guidelines 2025 [19] emphasize the need for a structured report to describe disease locations not only at diagnosis but also during follow-up. This approach ensures that the radiologist not only detects and characterize areas of disease involvement, but also comments on the potential resectability or unresectability of specific sites that may compromise the achievement of complete cytoreduction (R = 0) (Figure 3). This is particularly relevant in cases with the mesentery root involvement, diffuse carcinomatosis on the surface of the small intestine (resection of which would result in short bowel syndrome), diffuse involvement/infiltration of the stomach, duodenum, head of the pancreas, and finally involvement of the celiac axis and its branches (celiac lymph nodes may be removed) [22,23,24].

### 2.3. MRI

Although several ultrasound (US) diagnostic scoring systems with high sensitivity and specificity have been proposed in the literature to effectively stratify the risk of malignancy in ovarian lesions (see Section 2.1), up to 30% of adnexal masses remain indeterminate on US, posing significant challenges for treatment planning [25,26]. In this setting, contrast-enhanced magnetic resonance imaging (MRI) is considered the imaging modality of choice for the characterization of indeterminate ovarian lesions. MRI allows a more accurate assessment of lesions that cannot be adequately characterized on US, thereby lowering the level of suspicion and, consequently, reducing the number of unnecessary surgeries for benign lesions in asymptomatic patients. Reported performance metrics include a positive predictive value (PPV) of 71%, a negative predictive value (NPV) of 98%, and an overall diagnostic accuracy ranging from 83% to 93% for the detection of malignancy [25,27,28,29,30]. Moreover, the inherent high contrast resolution of MRI and its ability to characterize tissues enable a more accurate definition of lesion morphology. This advantage is further enhanced by the multiparametric and multiplanar nature of the technique, which allows comprehensive assessment of all lesion components, including areas that may not be accessible by transvaginal or transabdominal ultrasound [31]. In addition, diffusion weighted sequences (DWI) has proven to be more effective in predicting surgical outcome due to its capability to depict small peritoneal implants [32,33]. MRI also plays a key role in the evaluation of large pelvic masses of uncertain adnexal origin detected on US, as up to 10% of these lesions are ultimately found to be non-adnexal in nature and therefore reclassified [25].

As in ultrasound diagnostics, considerable efforts have been made to establish a standardized and universally understandable language for MRI characterization of adnexal masses—one that is reproducible, easily interpretable, and comprehensive for both lesion description and malignancy risk assessment. The first structured approach in this regard was the ADNEX MR model, proposed by Thomassin-Naggara et al. in 2013 [34].

#### 2.3.1. ADNEX-MR Scoring System

The “ADNEX-MR” Scoring System model was developed to provide a reproducible preliminary validation method for the use of pelvic MR in the characterization of adnexal masses which resulted “indeterminate” at ultrasound evaluation. In this study, the Authors made use of 1.5T MRI scanner with “phased-array” coils, after treatment of the patients with intravenous spasmolytic drug for better image resolution (in particular, glucagon), using the following protocol: axial and sagittal non-“fat-sat” T2-TSE sequences, T1 gradient-echo sequences with and without fat saturation, DWI in the axial plane with b levels up to 1000 s/mm^2^. Sequential T1 GRE perfusion sequences were acquired during contrast medium administration with a temporal resolution interval of 2.4 s for a total of 320 s, starting 10 s before contrast medium pier injection (2 mL/s); a delayed axial or sagittal T1 gradient sequence was also obtained. The purpose of the perfusion study was to compare myometrial enhancement with that of solid tissue within the adnexal lesion. In this context, solid tissue was defined as the presence of vegetations, solid areas, or irregular thickening of the septa or walls [35]. Specifically, two ROIs (region of interest) were placed: one in the outer myometrium and the other in the enhancing portion of intralesional solid tissue, with the purpose of making a comparison between two time-signal curves. A gradual increase in the signal intensity (enhancement) of intralesional solid tissue without a well-defined peak corresponds to a type 1 curve. A moderate initial increase in the solid tissue signal relative to that of the myometrium followed by a plateau corresponded to a type 2 curve. A type 3 curve was defined when the peak of solid tissue enhancement in the lesion occurred earlier and more steeply than that of the myometrium.

Despite the numerous imaging features evaluated in ovarian lesions, the criteria selected for lesion classification included: presence or absence of solid tissue, solid tissue signal in T2w sequences and in diffusion (DWI) sequences, type of enhancement curve, and presence of peritoneal implants (Table 2).

According to the above-described ADNEX model, ovarian lesions can be classified with the following scores:

1. Absence of ovarian lesions
2. Benign lesions: lesions with homogeneous content (serous, blood, fat) and the absence of wall enhancement and/or with hypointense solid tissue signal in T2 sequences and at high b values
3. Probably benign lesions: absence of solid tissue or solid tissue with type 1 enhancement curve
4. Indeterminate lesions: presence of solid tissue with type 2 curve
5. Likely malignant lesions: type 3 enhancement curve and presence of peritoneal implants

The ADNEX model was found to predict the risk of malignancy with more than 90% accuracy. According to the results obtained, the type 3 curve is the most predictive criterion for malignancy, along with the presence of irregular septa and peritoneal implants; conversely, in Score 2, the risk of malignancy is extremely low (<2%) [34].

#### 2.3.2. O-RADS MRI Score

The O-RADS (Ovarian-Adnexal Reporting and Data System) MRI score represents the most recent and widely accepted system for stratifying the risk of malignancy in ovarian lesions, developed by an international multidisciplinary panel of experts. By estimating the probability of malignancy in ovarian lesions, it aims to improve diagnostic accuracy, facilitate clearer interdisciplinary communication, and ultimately promote optimal patient management [27]. The O-RADS MRI system represents an evolution of the ADNEX MR model [34] described in the previous section, and it benefits from an overall diagnostic accuracy of 92%, associated with a sensitivity of 93% and specificity of 91%, PPV of 71% and NPV of 98% [25,26,36]. The first step in the O-RADS model building process involves the use of correct and standardized terminology. Thereafter, a lesion is first defined as a non-physiologic finding. Its features and the corresponding radiologic definitions are then carefully characterized, for instance the type of signal intensity (homogeneous/inhomogeneous and hypointense/intermediate/hyperintense), the type of lesion (cystic/solid) and the fluid content (simple/hemorrhagic/proteinaceous/fat). Solid components (such as papillary projections, mural nodules, irregular soft-tissue patches, or solid portions) are defined as areas showing contrast enhancement, whereas non-solid elements include clots, debris, fibrin, and similar materials. For a more comprehensive overview of terminology, please refer to the following articles [27,37,38].

Acquisition protocol.

**Figure 4:**
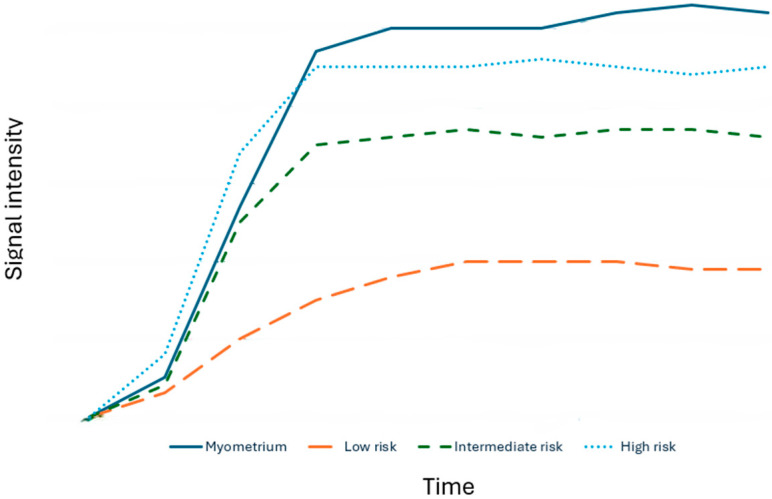
Signal–time curves used in the O-RADS MRI model to stratify the malignancy risk of ovarian lesions. The comparison between lesion enhancement and the myometrial reference curve allows classification into low-, intermediate-, and high-risk categories, contributing to standardized lesion risk assessment.

The examination protocol includes the acquisition of non-FS sagittal T2w sequences (≤4 mm), axial T2w sequences (≤3 mm), in-phase and out-phase T1-weighted sequences (≤4 mm), DWI in the axial plane (≤4 mm; b value > 1000 s/mm^2^), and finally post-contrast T1-weighted sequences. In this regard, along the lines of the ADNEX model, a dynamic study (DCE) T1-weighted sequences characterized by a resolution time of 15 s and a slice thickness of 3 mm is proposed, with acquisition beginning 30 s after contrast medium injection and lasting for 4 min. This study allows calculation of signal/time curves by placing ROIs on the solid tissue of the lesion and on the myometrium, being careful not to place the latter on the vessels. Specifically, the signal/time curve can be of three types when compared with the myometrial curve (Figure 4):

1. Type 1 (low risk): slower increase in signal intensity (enhancement) of solid tissue than that of the myometrium, without a peak or plateau
2. Type 2 (intermediate risk): moderate initial increase in solid tissue signal with a slower or equal slope to that of the myometrial, followed by a plateau
3. Type 3 (high risk): pronounced signal increase with an early peak compared with that of myometrium

In case a perfusion study (DCE with resolution < 15 s) cannot be acquired, this can be replaced by ultrafast grandient-echo sequences at 30–40 s after contrast medium injection (thickness ≤ 3 mm), thus allowing an alternative comparison with myometrium. Additionally, in patients who have undergone a hysterectomy, the radiologist can evaluate the morphology of the signal/intensity curve concerning the solid portions with contrast enhancement, taking into account the curve morphology (with or without a plateau).

CLASSIFICATION.

O-RADS MRI Score 0.

Lesions that cannot be properly evaluated on MRI examination fall into this category; for example, when some portions of the lesion are not included in the study volume, when all sequences could not be acquired, or when artifacts compromise the quality of the examination.

O-RADS MRI Score 1.

Findings of normality. In premenopausal women, follicles, corpora lutea, and hemorrhagic cysts up to a maximum size of 3 cm are classified as O-RADS 1; therefore, these findings are not considered pathological. In postmenopausal women, some follicles may still be present in the ovarian parenchyma, and if the radiologist defines these findings in the “normal range”, they will also receive a score of 1, thus not considering them lesions.

O-RADS MRI Score 2 (risk of malignancy < 0.5%).

In both pre- and postmenopausal women, it is important to apply this score only in those formations considered to be lesions, as follows:

- Unilocular cystic lesions with homogeneous fluid content, without wall enhancement and without solid tissue
- Unilocular cysts with uncomplicated fluid or endometrioid content, with mild wall enhancement but without intracystic solid portions (e.g., endometrioma)
- Adipose-content lesions without intralesional solid tissue; in this case, the adipose tissue shows hyperintense signal in T2- and T1-weighted sequences, with signal drop in fat-sat sequences. It is necessary to distinguish Rokitansky nodule, which may show enhancement, from solid tissue (e.g., mature teratoma).
- Solid lesions with homogeneously hypointense signal both in T2w and DWI sequences at high b-values, regardless of the type of enhancement after mdc (e.g., ovarian fibroma)
- Fallopian tubes dilated by simple fluid, with mild and subtle wall enhancement in the absence of solid tissue
- Para-ovarian cysts at any type of fluid content, which may or may not show wall enhancement, without intralesional solid tissue.

O-RADS MRI Score 3 (risk of malignancy ≈ 5%).

- Unilocular cysts (proteinaceous/hemorrhagic/mucinous fluid content) with wall enhancement but without solid tissue
- Multilocular cysts at any type of fluid content, with thin septa that may show enhancement and absence of solid tissue
- Lesions with solid tissue (excluding solid lesions described in score 2) that show low-risk enhancement curve (type1).
- Fallopian tubes with non-simple fluid content, thickened walls, no solid tissue.

O-RADS MRI Score 4 (risk of malignancy ≈ 50%).

**Figure 5:**
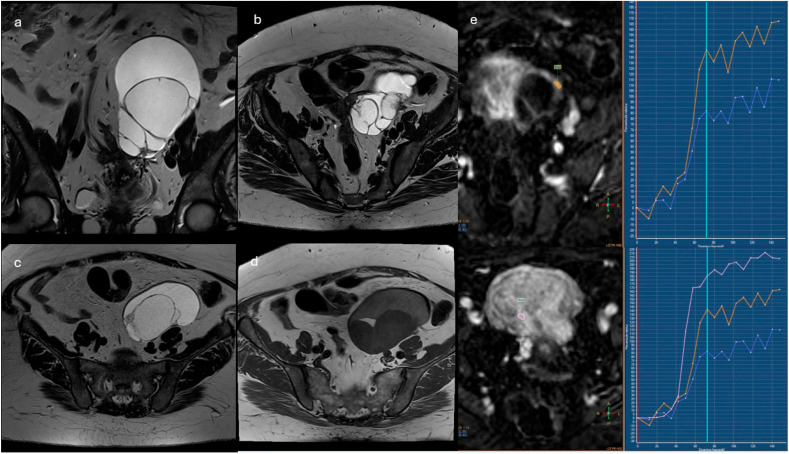
Left adnexal multiloculated cystic mass (**a**–**d**), with different signal intensity of the fluid in the loculi, especially in T1w sequences. In DCE-perfusion ROI were placed in thickened septa and wall ((**e**), orange and blue curves) and in the myometrium ((**e**), pink curve). The lesion was classified as O-RADS MRI Score 4 and resulted in mucinous cystoadenofibroma.

- Lesions with solid tissue (excluding solid lesions described in score 2) showing type 2 enhancement curve (intermediate risk; Figure 5)
- Solid lesions showing enhancement < myometrial at 30–40 s, if perfusion study is not available
- Lesions with lipid components and solid tissue with enhancement

O-RADS MRI Score 5 (risk of malignancy ≈ 90%).

**Figure 6:**
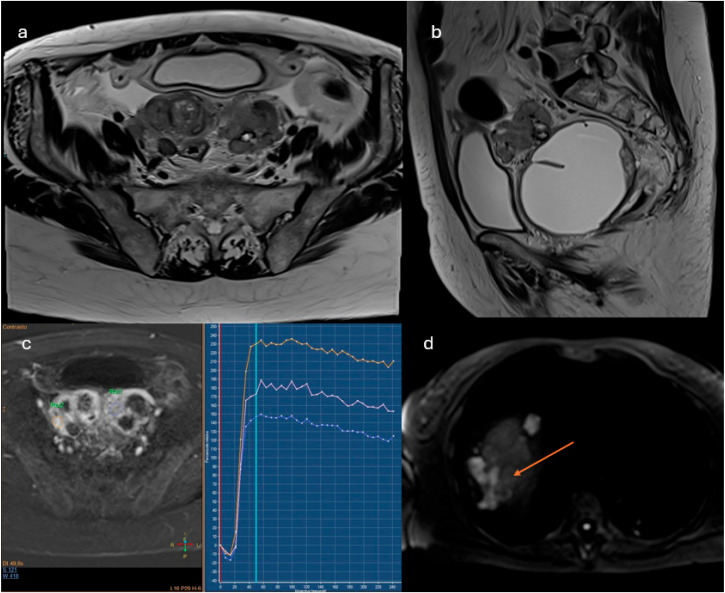
Bilateral adnexal masses (**a**,**b**). In DCE-perfusion ROI were placed in two different sites of the solid tissue ((**c**), orange and blue curves); the orange curve resulted “high risk curve” if compared to the curve of the myometrium ((**c**), pink curve): O-RADS 5. The study was extended at the upper abdomen with DWI, where peritoneal implants in subphrenic space were detected ((**d**), arrow).

- Lesions showing a type 3 enhancement curve (high risk)
- Solid lesions showing enhancement > myometrium at 30–40 s, if perfusion study is not available
- Lesions associated with the presence of peritoneal implants and/or secondary disease localization (Figure 6)

The examination protocol involves the acquisition of non-fat saturated sagittal T2w sequences (≤4 mm), axial T2w sequences (≤3 mm), in-phase and out-phase T1-weighted sequences (≤4 mm), DWI in the axial plane (≤4 mm; b value > 1000 s/mm^2^), and finally post-contrast T1-weighted sequences. In line with the ADNEX model, a dynamic contrast-enhanced (DCE) T1-weighted acquisition is recommended, with a temporal resolution of 15 s and a slice thickness of 3 mm; the dynamic series should begin 30 s after contrast medium injection and lasting for 4 min This acquisition enables the generation of signal–time curves by placing ROIs on the solid tissue of the lesion and on the myometrium, being careful not to place the latter on the vessels. Specifically, three enhancement curve types can be identified when compared with the myometrial reference curve (Figure 4):

1. Type 1 (low risk): delayed and slower enhancement of the solid component relative to the myometrium, without a peak or plateau
2. Type 2 (intermediate risk): moderate early enhancement of solid tissue with a slower or equal slope to that of the myometrial, followed by a plateau
3. Type 3 (high risk): pronounced signal increase with an early peak occurring before the myometrial peak

In case a high-temporal-resolution perfusion study (DCE < 15 s) cannot be performed, an ultrafast gradient-echo acquisition obtained 30–40 s after contrast injection (≤3 mm) may be used as an alternative, thus allowing a different type of comparison with myometrium. Additionally, in patients who have undergone hysterectomy, the radiologist can evaluate the morphology of the signal/intensity curve concerning the solid portions with contrast enhancement, taking into account the curve morphology (with or without a plateau).

### 2.4. ^18^F-FDG PET/CT

^18^F-fluorodeoxyglucose positron emission tomography/computed tomography combines metabolic and anatomical information, providing valuable insights into tumor biology and disease distribution. Although PET/CT is not routinely recommended for primary diagnosis or initial staging of ovarian cancer—mainly due to limited sensitivity for small peritoneal implants and false positive results in inflammatory conditions (such as endometriosis and hydrosalpinx)—it plays a crucial complementary role in specific clinical settings [39]. PET/CT is highly effective in detecting recurrent or residual disease, particularly when serum CA-125 levels rise but conventional imaging (CT or MRI) is inconclusive. Its ability to distinguish active disease from post-therapeutic changes or fibrosis makes it a powerful tool for response assessment and follow-up [40,41]. PET/CT can also contribute to preoperative evaluation, helping to identify extra-abdominal metastases (such as in lymph nodes, pleura, or liver parenchyma) that may alter surgical planning [42]. Quantitative metabolic parameters—such as SUVmax, metabolic tumor volume (MTV), and total lesion glycolysis (TLG)—have been investigated as prognostic biomarkers, correlating with tumor aggressiveness, chemotherapy response, and survival outcomes [43].

## 3. New Perspectives and AI in Ovarian Cancer

### 3.1. O-RADS MRI/ADC Score

Several studies have attempted to implement the O-RADS MRI Score, particularly category 4, where the positive predictive value (PPV) range for malignancy is highly variable (5–90%), highlighting the potential usefulness of the apparent diffusion coefficient (ADC) map of both cystic and solid components [44,45,46]. These studies have demonstrated that signal intensity in diffusion sequences, along with corresponding ADC values, can influence the radiological classification within the traditional O-RADS framework. This adjustment allows the upgrading or downgrading of lesions between scores 3–4 and 4–5, thereby reducing the large variability inherent in category 4, as described in the “O-RADS MRI/ADC Score” proposed by Manganaro et al. [46]. This investigation also revealed a significant correlation between ADC values and the histologic variant of the tumor, with borderline tumors exhibiting higher mean ADC values than low-grade and high-grade serous carcinomas.

### 3.2. NON- CONTRAST- MRI Score

**Figure 7:**
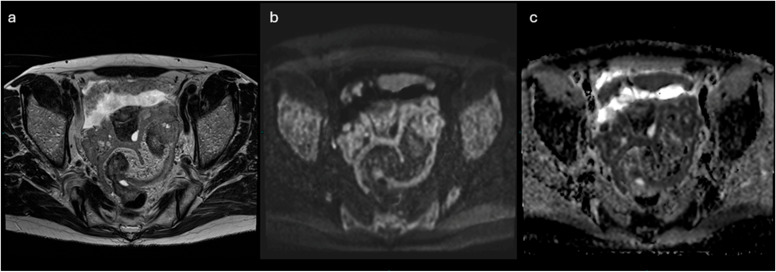
MRI scan of the pelvis of Patient of IMG. 1. The solid tissue in adnexal region shows intermediate signal intensity on T2-weighted sequences (**a**) and signal restriction on diffusion imaging (**b**,**c**), such as the solid implants along the peritoneum and the pelvic wall. See also the ascites in T2 sequences (**a**). Non-contrast MRI score: 5.

A retrospective study by Sahin et al. [47] evaluated the feasibility of characterizing and stratifying the malignancy risk of adnexal lesions without contrast medium administration. This was achieved by constructing a 5-point score based on morphologic and qualitative diffusion-weighted imaging (DWI) characteristics, redefining the term “solid tissue” as “tissue component showing intermediate signal on T2w sequences, low signal on T1w and diffusion restriction in DWI(ADC) sequences” (Table 3; Figure 7). The “Non-contrast MRI Score” has proven to be a reliable and reproducible tool, demonstrating high diagnostic accuracy (94.2%), excellent levels of sensitivity (84.9%) and specificity (95.9%), and substantial inter-reader agreement. It, therefore, represents a potential alternative to the O-RADS MRI score in situations where contrast medium administration is not recommended, such as in cases of severe allergic reactions to gadolinium-based agents, pregnancy status or severe nephropathy. The main limitation is the lack of external validation by a prospective, multicenter study; however, such validation is currently ongoing and has shown promising preliminary results.

### 3.3. PET-MRI

Although PET-MRI has been clinically available from a decade, its adoption remains limited compared to the widespread use of [18F] FDG PET-CT. This is due to a lack of standardised imaging protocols and workflow harmonisation, as well as costs, which hinder broader clinical use [19]. In ovarian cancer, PET-MRI has demonstrated a better diagnostic performance over PET-CT and diffusion-weighted MRI (DWI-MRI) in peritoneal staging at initial diagnosis (*p* = 0.001), though no significant difference was found after chemotherapy [48,49]. Preliminary evidence also supports PET-MRI’s utility in detecting recurrence of gynecologic malignancies; however improving diagnostic randomized studies are needed to fully define its added value in routine clinical practice [50].

In Table 4, the main imaging modalities for ovarian carcinoma are summarized, along with their key characteristics, primary applications, strengths, and limitations. The following chapter will address the role of Artificial Intelligence and how it can potentially enhance imaging evaluation in ovarian cancer.

### 3.4. Artificial Intelligence (AI)

Artificial intelligence (AI) is playing an increasingly important role in medical diagnostics, including imaging evaluation of ovarian cancer. This technology is intended to improve the accuracy, speed, and efficiency of diagnostic workflows, particularly in the analysis of medical images obtained by computed tomography (CT), magnetic resonance imaging (MRI), or ultrasound (US) [51]. High-grade serous carcinoma is now well recognized to exhibit substantial inter- and intra-tumoral heterogeneity at genomic and molecular levels, driven by the spatial distribution of tumor subclones and their evolving mutational and gene-expression profiles over time [52]. This statement is equally true in residual lesions after treatment, due to therapy-induced chemoresistance [53]. Radiomics, combined with machine-learning (ML) and deep-learning (DL) algorithms, enables the quantitative assessment of different tissue microarchitectures by extracting high-dimensional data from small imaging volumes, thus allowing the distinction of different “microenvironments” and different molecular profiles both within the primary lesion and across various sites of disease spread [54,55,56,57,58]. Non-invasive characterization of tumor heterogeneity introduces the concept of the “virtual biopsy”, which, in association with genomic, pathologic, clinical and laboratory variables, has the potential to improve understanding of tumor evolution and assist in selecting the most appropriate therapeutic strategy for each patient.

**Figure 8:**
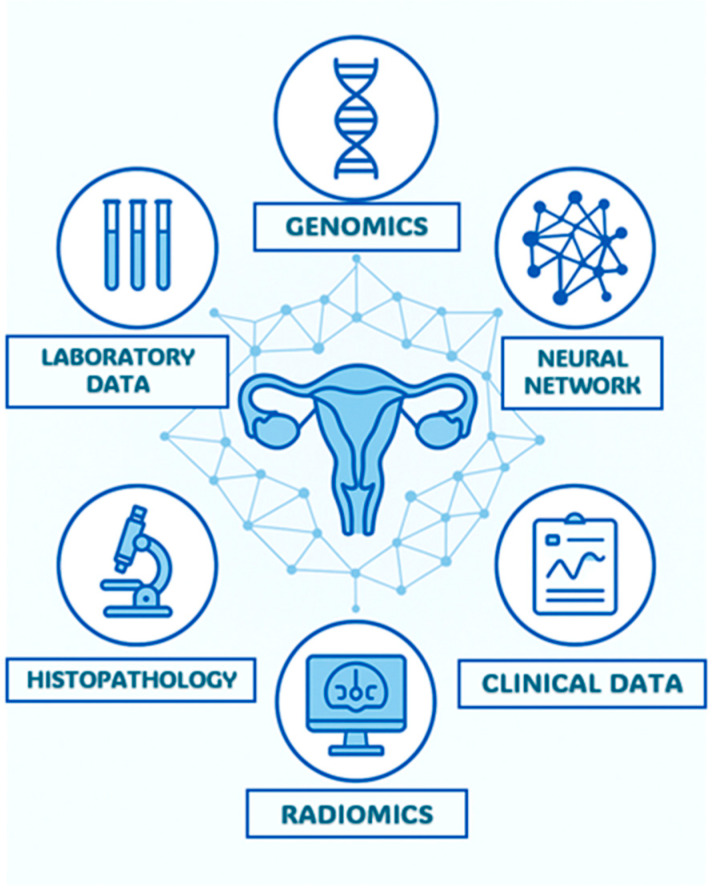
Schematic representation of artificial intelligence (AI) models integrating heterogeneous data sources—such as imaging features, clinical parameters, and molecular or histopathological information—to improve risk stratification, prognostic assessment, and personalized management of patients with ovarian cancer.

The main areas in which AI is influencing ovarian cancer imaging include:

1. Classification of lesions: although histopathological examination after biopsy remains the gold standard for distinguishing benign from malignant lesions, several studies suggest that deep learning (DL) algorithms—a subcategory of machine learning (ML)—have the potential to classify ovarian lesions with accuracy levels comparable to those achieved by radiologists. However, many of these reports provide limited methodological detail, often omitting the specific DL or ML architectures employed and lacking systematic comparisons between different models. These limitations impede the identification of the most effective techniques and reduce the generalizability of the available evidence, thereby limiting both scientific rigor and clinical applicability. Li et al. [59] reported a radiomics model capable of differentiating malignant and benign ovarian lesions in CT images of approximately 143 patients. Good diagnostic accuracy has also been found in radiomics applied to MRI, as described by Saida et al. [60] and Wang et al. [56]. A recent multicenter study assessed Meta’s Segment Anything Model (SAM) and a DenseNet-121 deep learning (DL) model for the classification of 621 ovarian lesions in 532 MRI-examined patients. The study assessed the accuracy of automatic SAM segmentation compared with manual segmentation and the diagnostic performance of the DL model and radiologists (using O-RADS MRI and histopathology as reference). No significant differences emerged between manual and SAM-based segmentation (AUC 0.83 vs. 0.79), nor between DL and radiologists’ classifications (AUC 0.79 vs. 0.84–0.86, *p* > 0.05). SAM segmentation reduced processing time by approximately 4 min per case without compromising diagnostic accuracy [61]. Although a direct comparison between machine learning (ML) and deep learning (DL) models would be highly desirable, the current literature is characterized by heterogeneous datasets, non-uniform outcome measures, and variable validation strategies, which preclude meaningful head-to-head comparisons. Addressing this limitation should be a priority for future multicenter and benchmarking studies.
  In the field of ultrasonography recent studies have demonstrated levels of diagnostic accuracy by DL algorithms comparable to O-RADS-US and experienced sonographers [62,63]. However, a recent meta-analysis on performance of radiomics in ultrasound describes how the comparison with the reference IOTA-ADNEX model has not been sufficiently investigated. Moreover, the number of studies present in the literature is few, lacks external validation, and includes small sample sizes [64].

2. Prediction of genetic alterations: as already well known, in patients diagnosed with ovarian cancer it is critical to establish whether the BRCA-1 and BRCA-2 genes are mutated, since BRCA-mutated tumors are associated with increased chemosensitivity to platinum-based drugs resulting in increased PFS. [65] Despite Meier et al. found no significant correlation between radiomic features and BRCA mutational status [66], other authors were able to predict Ki-67 status by analyzing radiomic features derived from PET-CT images [67].
3. Prediction of disease spread at diagnosis: AI can automate the process of image segmentation, i.e., the isolation and analysis of suspicious areas in medical images. This segmentation allows radiologists to focus more accurately and quickly on potentially pathological areas. Indeed, although CE-CT is the gold standard for staging ovarian cancer, it has accuracy limitations in identifying small peritoneal implants (<1 cm) and localizations in specific areas such as the small bowel and mesentery. In addition, lesions are often “unmeasurable” according to RECIST 1.1 criteria [68]. Several studies describe how AI can predict the presence of peritoneal carcinosis and lymph node metastasis in HGSOCs on both CT and MR imaging by integrating radiomics with both clinical and laboratory factors such as age and CA-125 blood-levels [69,70,71].
4. Prediction of treatment response: the prediction of treatment response according to radiomics models deeply traces the analysis of the previously described intrinsic heterogeneity of the tumor and various microenvironments. It is evident that different “subclones” of tumor tissue exhibit varied responses to different drugs in relation to their histological and molecular features. Indeed, it has been shown that while the number of disease localizations at diagnosis correlates significantly with treatment response, there is no correlation between disease volume and therapy response [72]. Conversely, when combined with clinical and laboratory data, radiomic biomarkers have been found to accurately predict response to neoadjuvant chemotherapy (NACT) [72,73]. Similarly, some studies based on ML and DL algorithms seem to be able to predict the probability of platinum-resistance of high-grade serous carcinoma [54,74]. Consequently, even in post-NACT imaging re-evaluations, any residual tumor and/or new disease localization can be accurately characterized by describing the microarchitecture and estimating the “subclone” of origin. The same applies to patients who are candidates for immunotherapy, in whom reduced intratumoral heterogeneity seems to be associated with better response [75].
5. Prediction of Risk of Recurrence: Several studies have focused on estimating progression-free survival (PFS) and overall survival (OS) in order to identify patients at higher risk of recurrence [76]. Some investigations have demonstrated a significant relationship between radiomic features in CT imaging and OS, specifically showing that lower tumor heterogeneity is associated with improved OS [66,77]. Similarly, other studies have examined the relationship between CT radiomic features of ovarian masses [78,79] and peritoneal implants [77] with PFS, which has also been found to correlate with tissue heterogeneity. Rizzo et al. [78] demonstrated that three radiomic variables—specifically, the gray level run length matrix (GLRLM), 3D morphological features, and the gray level co-occurrence matrix (GLCM)—are significantly associated with disease progression at 12 months. According to Zagari et al., among the variables considered in their study that significantly correlated with PFS, shape and density appeared to have the strongest correlation [80]. Furthermore, high tumor tissue heterogeneity has been associated with an increased risk of incomplete surgical resection (R≠0) in non-BRCA-mutated patients [66]. Conversely, Vargas et al. reported that lower heterogeneity values were associated with greater surgical resectability [77] and, consequently, with higher OS and PFS values [5]. The integration of PET radiomic features with CT features, together with clinical variables, have been shown to further improve prognostic accuracy compared to models based solely on CT imaging [81].
  Even in the context of MRI, a nomogram based on radiomic features derived from MRI images, combined with clinical variables, has shown a good ability to identify patients at risk of disease recurrence [82].

6. Integration with Other Data Sources: AI can combine information from different diagnostic modalities (e.g., imaging, clinical history and laboratory results) to provide a more comprehensive and precise overview of the patient’s condition. As previously mentioned, most of the studies cited have incorporated alongside imaging features clinical variables such as age, FIGO stage, serum CA-125 levels, and the presence of residual tumor. The integration of such data into ML and DL algorithms is essential for obtaining more accurate information and achieving higher levels of diagnostic precision (Figure 8).

Despite these interesting potential applications, several important limitations remain, largely due to the lack of standardization in acquisition protocols, image segmentation, and radiomic features extraction and analysis. These issues reduce the reproducibility of results, which are often derived from retrospective studies [51,83]. At present, different AI models are used with various software and without standardized acquisition protocols. In addition, although most studies involved internal validation, the absence of external validation at several centers undermined the credibility and generalizability of the models [84]. Radiomic features can vary considerably depending on imaging quality, tumor location, and the technology employed; therefore, AI must be capable of handling this variability to be effective in diverse clinical settings. Additionally, the “black box” nature of certain neural networks can make it challenging to understand how an algorithm reaches a specific conclusion. Other limiting factors include financial costs, processing time, and the current need for manual adjustments, all of which hinder the AI integration into clinical practice. Consequently, although radiomics analyses based on US, CT and MRI have shown promising results in terms of differential diagnosis and prognostic prediction advancements, the incorporation of AI into ovarian cancer imaging still requires rigorous clinical validation and close supervision by healthcare professionals.

## 4. Conclusions

Ovarian cancer remains a leading cause of gynecologic cancer mortality, primarily due to late-stage diagnosis and treatment resistance. Imaging plays a central role throughout the diagnostic and therapeutic pathway. Ultrasound is the first-line modality for adnexal mass evaluation, with IOTA and O-RADS US systems providing standardized risk assessment. MRI serves as a crucial second-line technique, offering superior tissue characterization and high diagnostic accuracy, particularly in indeterminate or high-risk cases. The ORADS-MRI score represents the most widely accepted system for stratifying malignancy risk in ovarian lesions; however, new risk stratification systems have been proposed by many authors to further improve specificity or to allow assessment when contrast medium injection is not recommended. CT remains essential for disease staging and surgical planning, while PET-CT is valuable for detecting extra-abdominal spread and recurrence. Despite technological progress, standardized imaging protocols for follow-up are still lacking.

Emerging applications of artificial intelligence and radiomics show promising potential for improving lesion characterization, prognostic prediction, and treatment monitoring. However, their routine clinical adoption requires large, prospective and multicenter validation studies to ensure robustness, reproducibility, and safety since shortcomings persist in existing studies in various fields.

Future research should prioritize multicenter collaborations and the development of large, standardized imaging repositories that integrate clinical and molecular data. Harmonization of acquisition protocols, open validation frameworks, and the development of hybrid models that combine radiological expertise with AI-driven analytics are crucial steps toward clinically reliable, reproducible, and interpretable diagnostic tools. Ultimately, the convergence of advanced imaging, standardized reporting, and AI-assisted analysis holds the potential to personalize risk assessment and improve the management of ovarian cancer, but its successful translation into clinical practice will depend on rigorous methodological validation and interdisciplinary integration.

## Figures and Tables

**Table 1:** O-RADS US v2022 [17].

0	Incomplete Evaluation [N/A]	N/A
1	Normal Ovary [N/A]	Follicle defined as a simple cyst ≤ 3 cm; Corpus Luteum ≤ 3 cm
2	Almost Certainly Benign [<1%]	Simple cyst ≤ 3 cm
		Simple cyst > 3 cm to 5 cm
		Simple cyst > 5 cm but <10 cm
		Classic Benign Lesions (dermoid, endometrioma, etc…)
		Non-simple unilocular cyst, smooth inner margin ≤ 3 cm
		Non-simple unilocular cyst, smooth inner margin > 3 cm but <10 cm
3	Low Risk Malignancy [1–<10%]	Unilocular cyst ≥ 10 cm (simple or non-simple)
		Typical dermoid cysts, endometriomas, hemorrhagic cysts ≥ 10 cm
		Unilocular cyst (any size) with irregular inner wall < 3 mm height
		Multilocular cyst < 10 cm, smooth inner wall, CS = 1–3
		Solid smooth, any size, CS = 1
4	Intermediate Risk [10–<50%]	Multilocular cyst, no solid component, ≥ 10 cm, smooth inner wall, CS = 1–3
		Multilocular cyst, no solid component, any size, smooth inner wall, CS = 4
		Multilocular cyst, no solid component, any size, irregular inner wall and/or irregular septation, any color score
		Unilocular cyst with solid component, any size, 0–3 papillary projections, CS = any
		Multilocular cyst with solid component, any size, CS = 1–2
		Solid smooth non- shadowing, any size, CS = 2–3
5	High Risk [≥50%]	Unilocular cyst, any size, ≥ 4 papillary projections, CS = any
		Multilocular cyst with solid component, any size, CS = 3–4
		Solid smooth, any size, CS = 4
		Solid irregular, any size, CS = any
		Ascites and/or peritoneal nodules

**Table 2:** ADNEX-MR Scoring System model [34].

ADNEX MR SCORE	Criteria
1. No mass	No mass
2. Benign mass	Purely cystic mass Purely endometriotic mass Purely fatty mass Absence of wall enhancement Low *b* = 1000 s/mm^2^–weighted and low T2-weighted signal intensity within solid tissue
3. Probably benign mass	Absence of solid tissue Curve type 1 within solid tissue
4. Indeterminate MR mass	Curve type 2 within solid tissue
5. Probably malignant mass	Curve type 3 within solid tissue Peritoneal implants

**Table 3:** Non-Contrast MRI Score [47].

Non-Contrast MRI Score	Definition	MRI Features
Score 1	No mass	No adnexal mass is demonstrated in pelvic MRI study
Score 2	Benign/likely benign	Radiologically characterized with radiological diagnosis (e.g., endometrioma, dermoid, fibroma)
Score 3	Indeterminate	Not classified in other scores; it may have a solid appearing component without reaching criteria for solid tissue
Score 4	Suspicious for malignancy	Solid tissue criteria reached
Score 5	Highly suspicious for malignancy	Solid tissue criteria reached and presence of - Peritoneal implants - Lymphadenopathy and/or - Ascites in the presence of solid tissue, after benign diagnoses are excluded

**Table 4:** Comparison of different imaging modalities in ovarian cancer.

Modality	Main Uses	Strengths	Limitations
Transvaginal Ultrasound (TVUS)	First-line evaluation of adnexal masses. Assessment of morphology and vascularization.	Widely available, non-invasive, no radiation. High-resolution for pelvic organs.	Operator-dependent. Limited for staging and evaluation of peritoneal spread.
Computed Tomography (CT)	Staging (especially peritoneal, nodal, and distant metastases). Preoperative planning.	Good overview of abdomen and pelvis. Useful for surgical planning and monitoring recurrence.	Limited soft tissue contrast. Poor detection of small peritoneal implants (<1 cm).
Magnetic Resonance Imaging (MRI)	Characterization of indeterminate masses. Local staging. Evaluation of complex cystic lesions.	Excellent soft tissue contrast. No radiation. Functional imaging (DWI) adds value in lesion analysis.	More expensive, time-consuming. Contraindicated in patients with certain implants.
FDG-PET/CT	Detection of recurrence, metastases. Useful in equivocal cases.	Functional and anatomical data. High sensitivity in detecting active disease and distant spread.	Limited role in primary diagnosis. False positives in inflammation/endometriosis. Costly.
PET/MRI (emerging)	Research setting. Combines metabolic and high-resolution anatomic data.	Potentially best of both PET and MRI. Promising for advanced imaging and radiomic studies.	Limited availability. High cost. Not yet widely implemented in clinical practice.

## Data Availability

No new data were created or analyzed in this study.

## Abbreviations

OC	Ovarian cancer
US	Ultrasound
CT	Computed Tomography
CE-CT	Contrast-enhanced CT
MRI	Magnetic Resonance Imaging
ROI	Region of Interest
PPV	Positive Predictive Value
NPV	Negative Predictive Value
PET	Positron Emission Tomography
AI	Artificial Intelligence
ML	Machine Learning
DL	Deep Learning
PFS	Progression-Free Survival
OS	Overall Survival
